# Large offspring have enhanced lifetime reproductive success: Long‐term carry‐over effects of weaning size in gray seals (*Halichoerus grypus*)

**DOI:** 10.1002/ece3.10095

**Published:** 2023-06-06

**Authors:** Janelle J. Badger, W. Don Bowen, Cornelia E. den Heyer, Greg A. Breed

**Affiliations:** ^1^ Department of Biology and Wildlife University of Alaska Fairbanks Fairbanks Alaska USA; ^2^ Department of Fisheries and Oceans Canada Bedford Institute of Oceanography Dartmouth Nova Scotia Canada; ^3^ Institute of Arctic Biology University of Alaska Fairbanks Fairbanks Alaska USA; ^4^ Present address: Pacific Islands Fisheries Science Center National Oceanic and Atmospheric Administration Honolulu Hawaii USA

**Keywords:** capture‐recapture, carry‐over effects, grey seals, life history theory, mixed‐effects

## Abstract

An individual's size in early stages of life may be an important source of individual variation in lifetime reproductive performance, as size effects on ontogenetic development can have cascading physiological and behavioral consequences throughout life. Here, we explored how size‐at‐young influences subsequent reproductive performance in gray seals (*Halichoerus grypus*) using repeated encounter and reproductive data on a marked sample of 363 females that were measured for length after weaning, at ~4 weeks of age, and eventually recruited to the Sable Island breeding colony. Two reproductive traits were considered: provisioning performance (mass of weaned offspring), modeled using linear mixed effects models; and reproductive frequency (rate at which a female returns to breed), modeled using mixed effects multistate mark–recapture models. Mothers with the longest weaning lengths produced pups 8 kg heavier and were 20% more likely to breed in a given year than mothers with the shortest lengths. Correlation in body lengths between weaning and adult life stages, however, is weak: Longer pups do not grow to be longer than average adults. Thus, covariation between weaning length and future reproductive performance appears to be a carry‐over effect, where the size advantages afforded in early juvenile stages may allow enhanced long‐term performance in adulthood.

## Graphical abstract

An individual's size in early stages of life may be an important source of individual variation in lifetime reproductive performance, as size effects on ontogenetic development can have cascading physiological and behavioral consequences throughout life. Here, we found a positive correlation between an individual's natal length and two traits of their reproductive performance throughout adulthood, in a large‐sample of long‐lived grey seals (*Halichoerus grypus*). These results indicate a carry‐over effect of size advantages granted in early juvenile stages—the first such findings for marine mammals.

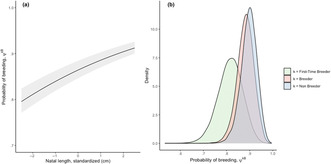

## 1 INTRODUCTION

Life history theory predicts that maternal fitness is maximized by the reproductive strategy, which results in the greatest number of offspring surviving to maturity, and subsequently producing large numbers of viable offspring themselves (Roff, 1992; Stearns, 1992). Variation in offspring quality may be influenced by parents through a myriad of pathways including the selection of safe and nutritious oviposition or birth sites, incubation behavior, food provisioning, defense of young, and investment in offspring size (Krist, 2011; Mousseau & Fox, 1998). These behaviors are costly, and reproductive strategies in long‐lived species will be driven by the relationship between offspring traits and environmental conditions that determines fitness (Allen et al., 2008; Mousseau & Fox, 1998; Smith & Fretwell, 1974).

Offspring size is one of the most important and well‐studied of these traits in evolutionary ecology, as natural selection on body size and size‐related traits is ubiquitous in nature (reviewed by Krist, 2011; Pettersen et al., 2015; Sogard, 1997). Within species, larger offspring typically outperform their smaller conspecifics, with higher survival rates to sexual maturity (e.g., plants: Stanton, 1984, marine invertebrates: Marshall et al., 2006; Moran & Emlet, 2001, gray seals: Bowen et al., 2015, lizards: Sinervo, 1990), enhanced resistance to starvation, environmental extremes, and predation (Sogard, 1997), increased metabolic efficiency (Pettersen et al., 2015), and higher reproductive performance found in some species (arthropods: Fox & Czesak, 2000, birds: reviewed in Krist, 2011, marine invertebrates: Marshall & Keough, 2008). Mothers may confer this advantage on their young either through a heritable genetic predisposition (possibly by choosing larger mates) or maternal effects such as nutrient transfer and protective behavior toward young (Bernardo, 1996; Mousseau & Fox, 1998).

The advantages of natal size are often pronounced in early stages of ontogeny, but may persist throughout life affecting reproduction and even the performance of the subsequent generation (Dias & Marshall, 2010; Lindström, 1999; Marshall et al., 2003). While many studies have confirmed the relationship between offspring size and survival, less is known about how the effects of natal size subsequently manifest in adults recruited to the breeding population. Even in mammals and birds where offspring are relatively large and individuals may be tracked, estimates of the effect of an individual's size when young on subsequent performance are available for only a few taxa (Clutton‐Brock, 1991; Crawley et al., 2017; Festa‐Bianchet et al., 2000; Fox & Czesak, 2000) and fewer still for natural populations. This knowledge gap is particularly apparent in long‐lived iteroparous animals, where it is difficult to track individuals’ reproductive performance and survival throughout an adulthood that may last decades. Offspring size effects on fitness would then be inaccurately estimated because key components of fitness are not measured at sufficient temporal scales (Marshall et al., 2003).

Reproductive and early life history traits can be considered aspects of either offspring or maternal phenotype, and their evolution will therefore depend on selection operating through both offspring and maternal components of fitness (Mousseau & Fox, 1998). Selection acts to maximize parental fitness, but offspring size also simultaneously influences offspring fitness. An individual's size when young may be an important source of individual variation in lifetime reproductive performance (individual quality), as size effects on ontogenetic development can have cascading physiological and behavioral consequences throughout life (Lindström, 1999). Size may mediate the expected trade‐off between growth, self‐maintenance, and mortality in early stages by increasing survival probabilities (avoiding starvation and predator escapement) and/or increasing foraging efficiency, allowing individuals to mature more quickly or invest in costly physiological functions that lead to greater lifetime reproductive output. This variation in individual quality is a key driver in natural selection and an important link between evolutionary and ecological processes (Bolnick et al., 2003, 2011; Cam et al., 2002; Gimenez et al., 2017; Lomnicki, 1978; Stover et al., 2012; Vindenes et al., 2008).

The extensively studied colony of gray seals (*Halichoerus grypus*) breeding on Sable Island, Nova Scotia, provides an excellent opportunity to explore the link between size when young and subsequent performance as adults. Gray seals are long‐lived (~40 years), iteroparous capital breeders in which females invest heavily into the survival of a single offspring over the course of a relatively short, intense lactation period lasting 16–18 days (Boness & James, 1979; Iverson et al., 1993). During the nursing period, mothers lose a third of their body mass on average (4.1 kg per day, Mellish et al., 1999) relying only on fat reserves to produce milk and maintain metabolism, while their pups typically more than triple their birth mass (2.8 kg per day, Bowen et al., 1992). At the end of lactation, females abruptly end care and return to the sea, which allows female reproductive expenditure to be accurately measured by the energy allocated to offspring (Bowen et al., 2007). In this system, offspring size is more variable than offspring number (twins are exceedingly rare), so offspring size (rather than litter size) is more subject to selection for maternal fitness.

Sable Island gray seal pup production (a proxy for population size) has increased dramatically over the past half century with near maximum population growth of 13% per year between the 1960s and late 1990s (Bowen, 2011) and a reduced rate of increase of 5%–7% per year since 2004 (den Heyer et al., 2017, 2021). Associated with declines in population growth, juvenile apparent survival to reproductive recruitment has decreased by more than half from an average of 74% in cohorts born 1985–1989 to 33% in cohorts born 1998–2002 (den Heyer et al., 2013). This decline appears to be size‐selective, with recent investigations finding that heavier and longer pups are more likely to recruit (Bowen et al., 2015). Apparent survival to recruitment increases asymptotically with mass at weaning, but monotonically with length at weaning (Bowen et al., 2015), indicating stabilizing selection for mass, but directional selection for larger early skeletal size. The survival advantage of larger skeletal size may be due to increased swimming speed and agility allowing greater foraging ability and predator escapement (Hindell et al., 1999; Sogard, 1997), though other physiological mechanisms cannot be ruled out. This size selection may be intensifying under density dependence, as young‐of‐the‐year gray seals now must make longer foraging trips and forage farther from haul‐out sites than older animals, which occupy foraging areas closer to rookeries (Breed et al., 2011, 2013), so larger bodied animals that can swim more efficiently may experience increased survival than shorter conspecifics.

Here, we use a 19‐year longitudinal dataset of repeated reproductive measurements from individually marked, known‐aged female gray seals whose lengths were measured after weaning (at roughly 4 weeks of age, hereby referred to as *weaning length*) to evaluate the influence of early size on subsequent long‐term reproductive success. As length is a better indicator than mass of overall skeletal size that may confer a more enduring advantage, we investigate whether variation in weaning length is associated with increased reproductive performance as adults, measured using two traits: reproductive rate and offspring size at weaning. If weaning length is positively associated with reproductive performance, we consider that support for a “bigger is better” hypothesis, in which maternal fitness is benefitted from bearing longer offspring that will subsequently have higher reproductive success. However, we discuss the probability that these effects represent a carryover of early body size rather than a life‐long size advantage, as neither weaning length nor weaning mass explains more than a few percent of the variation in adult length (Bowen et al., 2015), and thus larger (or smaller) pups do not necessarily grow into larger (or smaller) adults and larger pups express better lifetime reproductive performance even when they mature to be average sized adults.

## 2 METHODS

This study was conducted on Sable Island, Canada (43.93°N, 59.91°W), a partially vegetated sandbar on the Scotian Shelf roughly 160 km off the coast of Nova Scotia, during the 1998–2020 breeding seasons. The breeding season at this colony spans early December through early February, with 91.2% of pups born by mid‐January (Bowen et al., 2007; den Heyer et al., 2021). Sable Island supports the largest breeding colony of gray seals in the world with an estimated 87,500 pups (SE = 15,100) born on the island in 2016, comprising 80% of the total gray seal pup production in the Northwest Atlantic (den Heyer et al., 2021).

### 2.1 Data collection

Our 19‐year study (2002–2020) was conducted on a subset of female gray seals born on Sable Island from 1998 to 2002 that survived to recruit to the breeding colony, as part of a larger program led by the Department of Fisheries and Oceans, Canada (DFO). Individuals were marked at roughly 4 weeks old, shortly after weaning, with unique alpha‐numeric hot‐iron brands in each year 1998–2002. Pup body size growth during this postweaning fast in minimal, while the pups develop the infrastructure for diving along with other physiological mechanisms (Noren et al., 2008), so error due to the age of the pup relative to the time of measurement is minimal. Prior to marking, researchers recorded standard dorsal body length (to the nearest cm) of these individuals while they were sedated with diazepam (~0.4 mg/kg body mass, Sandoz Canada) to ensure accurate measurement standardized across individuals (Bowen et al., 2015). These permanent brands allowed reliable identification of individuals over the course of their lives. Females can recruit to the breeding population as early as 4 years old, but this is uncommon, and the average age of first reproduction is 6.5 ± 0.21 SE years for these cohorts (den Heyer et al., 2013) with 87% of females recruited at or before age 7 (Bowen et al., 2015). During each breeding season since 2002, teams of researchers conducted 5–7 roughly weekly censuses of branded females returning to the island to give birth and mate. Once sighted, branded individuals with dependent pups were visited daily but generally not disturbed. Prior to weaning, pups were sexed and marked with semipermanent, uniquely numbered tags in the hind flipper to ensure accurate identification after the marked female ended lactation and returned to sea, leaving her pup in the colony. Females attend their pups continuously throughout lactation. Therefore, once a pup was sighted alone, it was considered weaned and weighed to the nearest 0.5 kg.

The probability of observing a marked female during any given year includes both the probability the female is present, and the probability that she is detected given presence at the breeding colony. A recent analysis of this population indicated that, if a female rears a pup on the island, there is less than a 5% chance researchers will fail to detect her in at least one resighting census (Badger et al., 2020). Individuals that are not rearing pups can be skittish and may flee to the water, resulting in a lower sighting probability than females nursing and defending young. Gray seals are highly site philopatric, and once recruited to a breeding colony, will very rarely pup elsewhere (Bowen et al., 2015). Thus, we are able to reliably follow the reproductive history of individuals, and do not expect permanent emigration to other colonies to be a significant source of sighting error.

Individual sighting histories were collected from age at first reproduction (first sighting in breeding colony) until the most recent year of our study, 2020. Sighting histories of individuals were scored as a 0 (not sighted) or 1 (sighted) for each year 2002 to 2020. Females sighted in only one breeding season were omitted from this analysis to ensure that they had in fact recruited to the Sable Island breeding population and we have adequate data to estimate reproductive performance.

All procedures used on study animals were in compliance with applicable animal care guidelines of the Canadian Council on Animal Care and were approved by The Department of Fisheries and Oceans Animal Care Committee (Protocol numbers 98‐57 through 12‐08).

### 2.2 Statistical analysis

In this analysis, we were interested in understanding how a female's size during early life stages influences subsequent reproductive success once she has matured. To do this, we analyzed the effect of weaning length (*L*_*w*_, her length after weaning, but prior to independent foraging at approximately 4 weeks old) on her reproductive performance in adulthood, measured two ways: annual provisioning performance and reproductive frequency (both described below). We used generalized mixed effect additive and linear models to determine the effect of *L*_*w*_ on these traits, and accounted for imperfect detection in reproductive rate using a multistate capture–recapture model in a Bayesian framework (Gimenez et al., 2007; Kéry & Schaub, 2012; Lebreton et al., 2009).

#### 2.2.1 Modeling annual provisioning performance

During lactation, gray seal pups consume only milk provided by the female, and as capital breeders, females fast for the entire lactation period and provision pups exclusively from energy stores. Therefore, in our study, the body mass of a pup at weaning is a reasonable estimate of the energy (i.e., nutrients) transferred to young, and is of critical importance for pup survival (Bowen et al., 2015; Hall et al., 2001). We modeled the weaning mass of pup *j* born to female *i* in year *t* (mass_*j*,*t*_) as a linear mixed effects model with female experience (parity, i.e., *par*; because this effect tends to plateau, it was discretized into 1, 2, and 3+ parities), offspring sex, and a quadratic effect of standardized female age as covariates along with random individual and year intercepts:

$${\text{mass}}_{j,t}=\pi_{1}\cdot {\mathrm{age}}_{i,t}+\pi_{2}\cdot {\mathrm{age}}_{i,t}^{2}+\pi_{3,m}+\pi_{4}\cdot I\left({\mathrm{sex}}_{j,t}=\text{female}\right)+\alpha_{i}+\eta_{t}+\upsilon_{i,t}$$

where linear parameters are held in the vector ***π*** = {*π*_1_, *π*_2_, *π*_3_, *π*_4_} and represent linear and quadratic age effects, effect of female experience, and pup sex, respectively; and **I** signifies an indicator variable, and *m* denotes the parity group (1, 2, or 3+) of female *i* in year *t* so $m\in \left\{1,2,3\right\}$. *α*_*i*_ is the random effect of individual such that $\alpha_{i}∼N\left(0,\sigma_{\alpha }^{2}\right)$, *η*_*t*_ reflects the random year effect, where $\eta_{t}∼N\left(0,\sigma_{\eta }^{2}\right)$, and *υ*_*i*,*t*_ is the error term where $\upsilon_{i,t}∼N\left(0,\sigma_{\upsilon }^{2}\right)$.

We tested the effect of *L*_*w*_ on the history of her pup weaning masses comparing this null model to models including *L*_*w*_ as a linear term and a quadratic term (Table 1, Appendix A: Table A1). We also included a model in which the effect of *L*_*w*_ on offspring size varies with parity, such that the effect may diminish over time (Dias & Marshall, 2010). Models were fit using the lmer function in package lme4 (Bates et al., 2015), and support for model configurations was determined via likelihood ratio tests using the ANOVA function offered in R (R Core Team, 2020).

**TABLE 1:** Four competing linear mixed effects models to describe the effect of weaning length on her reproductive performance, measured as offspring mass.

Model	Form	AIC	LRT *p* value
Mod 0: Null	${\text{mass}}_{j,t}=\pi_{1}∙{\mathrm{age}}_{i,t}+\pi_{2}∙{\mathrm{age}}_{i,t}^{2}+\pi_{3,m}+\pi_{4}∙I\left({\mathrm{sex}}_{i,t}=\text{female}\right)+\alpha_{i}+\eta_{t}+\vartheta_{i,t}$	17,713	‐
Mod 1: Linear effect of weaning length	Mod 0 + π_5_·*L*_*M*,*i*_	17,702	*p* < .001
Mod 2: Quadratic effect of weaning length	Mod $0+\pi_{5}∙L_{M,i}+\pi_{6}∙L_{M,i}^{2}$	17,702	*p* = .176
Mod 3: Interactive effect with maternal experience	Mod $0-\pi_{3,m}+\pi_{5}∙L_{M,i}+\pi_{6}∙L_{M,i}∙I$(${\mathrm{par}}_{i,t}=2$)$+\pi_{7}∙L_{M,i}∙I$(${\mathrm{par}}_{i,t}=3$)	17,705	*p* = .602
Mod 4: Cohort effects	Mod $0+\pi_{5}∙L_{M,i}+\pi_{c}$, where $c\in \left\{1998,1999,2000,2001,2002\right\}$	17,707	*p* = .631

*Note*: Where mass_*i*,*t*_ is the mass of the weaned pup born to female *i* in year *t*. Parameters ***π*** = {*π*_1_, *π*_2_, *π*_3_, *π*_4_} reflect the quadratic age effect, effect of female experience, and pup sex, respectively, and $\pi \in \left\{\pi_{5},\pi_{6},\pi_{7}\right\}$ describe the effect of maternal weaning length, *L*_*w*_ under different models. *α*_*i*_ is the random effect of individual such that $\alpha_{i}∼N\left(0,\sigma_{\alpha }^{2}\right)$, *η*_*t*_ reflects the random year effect, where $\eta_{t}∼N\left(0,\sigma_{\eta }^{2}\right)$.

#### 2.2.2 Modeling reproductive rate

The second reproductive trait, reproductive rate, is defined as the probability an individual will return to the island to give birth in any given year.

We estimated the effect of a female's weaning length *L*_*w*_ on her reproductive rate by modeling her reproductive history as a Markov chain in a multistate capture–recapture modeling framework (Chambert et al., 2013; Gimenez et al., 2007; Kéry & Schaub, 2012; Lebreton et al., 2009). Between her first and last sightings on the island during our study, a female transitions among three reproductive states: initially a first time breeder *F* upon her first sighting, then switching between a breeder state *B*, or nonbreeder state *N*. An individual's state transitions from year *t* to *t* + 1 is modeled as a categorical trial with probabilities of transition *ψ* ^*ks*^ from state *k* to state *s*. Reproductive frequency is then defined as the probability of transition from any state *k* into the reproductive state *B* (*ψ* ^*kB*^). Females are necessarily undetected in state *N*, but observations of state *B* are governed by a detection probability. We used mixed effects logistic regression embedded in this multistate model to account for standardized female age, previous breeding state, and random individual and year effects in probability of breeding (*ψ* ^*kB*^):

$$\psi_{i,t}^{\mathrm{kB}}=\mu +\lambda_{1}∙{\mathrm{age}}_{i,t}+\lambda_{2}∙{\mathrm{age}}_{i,t}^{2}+\lambda_{3,k}+\beta_{i}+\theta_{t}+\omega_{i,t}$$

where parameters ***λ*** = {*λ*_1_, *λ*_2_, *λ*_3_} represent the quadratic age effect and the effects of the previous breeding state *k*, respectively, where parameters *λ*_3,*k*_ sum to zero. *β*_*i*_ is the random effect of individual such that $\beta_{i}∼N\left(0,\sigma_{\beta }^{2}\right)$, *θ*_*t*_ reflects the random year effect, where $\theta_{t}∼N\left(0,\sigma_{\theta }^{2}\right)$, and *ω*_*i*,*t*_ is the error term where $\omega_{i,t}∼N\left(0,\sigma_{\omega }^{2}\right)$.

Similar to above, we tested the effect of weaning length *L*_*w*_ on a female's reproductive rate by comparing this null model to models including *L*_*w*_ as a linear term and a quadratic term (Table 2). Furthermore, we included a model in which the effect of *L*_*w*_ on offspring size varies with parity, such that the effect may diminish over time.

**TABLE 2:** Four competing multistate mixed effects mark‐recapture models to describe the effect of weaning length on her reproductive performance, measured as reproductive rate.

Model	Form	WAIC	_*δ*_ WAIC
Mod 0: Null	$\psi_{i,t}^{\mathrm{kB}}=\mu +\lambda_{1}∙{\mathrm{age}}_{i,t}+\lambda_{2}∙{\mathrm{age}}_{i,t}^{2}+\lambda_{3,k}+\beta_{i}+\theta_{t}+\omega_{i,t}$	2503.1	14.8
Mod 1: Linear effect	Mod 0 + λ_5_·*L*_*M*,*i*_	2488.3	0
Mod 2: Quadratic effect	Mod $0+\lambda_{5}∙L_{M,i}+\lambda_{6}∙L_{M,i}^{2}$	2489.7	1.4
Mod 3: Interactive effect with maternal experience	Mod $0+\lambda_{5}∙L_{M,i}+\lambda_{6}∙L_{i}∙I$(${\mathrm{par}}_{i,t}=1$)	2498.0	9.7
Mod 4: Cohort effects	Mod $0+\lambda_{5}∙L_{M,i}+\lambda_{c}$, where $c\in \left\{1998,1999,2000,2001,2002\right\}$	2497.44	9.14

*Note*: Where $\psi_{i,t}^{\mathrm{kB}}$ is the probability that female *i* will be in a breeding state in year *t*. Parameters ***λ*** = {*λ*_1_, *λ*_2_, *λ*_3_,*λ*_4_} reflect the quadratic age effect and the effect of previous states, respectively, and $\lambda \in \left\{\lambda_{5},\lambda_{6}\right\}$ describe the effect of maternal weaning length, *L*_*w*_ under different models. *β*_*i*_ is the random effect of individual such that $\beta_{i}∼N\left(0,\sigma_{\beta }^{2}\right)$, *θ*_*t*_ reflects the random year effect, where $\theta_{t}∼N\left(0,\sigma_{\theta }^{2}\right)$.

Multistate models can also be used to detect a cost of reproduction (e.g., Badger et al., 2020; Beauplet et al., 2006; Chambert et al., 2013; Hernández‐Matías et al., 2011; Johns et al., 2018; Stoelting et al., 2015). A common approach is to determine whether breeding at time *t* negatively affects an individual's probability of surviving from time *t* to *t* + 1 or its probability of breeding at time *t* + 1. In the model used here, one way in which a cost of reproduction may be observed as a higher probability of transition *ψ* into a breeding state *B* from a nonreproductive state *N*, that is, *ψ* ^*NB*^ > *ψ* ^*BB*^.

A Bayesian approach was used for estimation and implemented in the software program JAGS 4.2.0 using the R interface rjags (Plummer, 2003, 2018; R Core Team, 2020). Parameters ***λ*** were assigned diffuse normal prior distributions *N* (0, 1000). Random year term *θ* was specified hierarchically following a normal distribution, $\theta_{t}∼N\left(0,\sigma_{\theta }^{2}\right)$, and individual terms *β*_*i*_ were pulled from a $N\left(0,\sigma_{\beta }^{2}\right)$. We specified a Unif(0,10) prior for *σ*_*θ*_ and *σ*_*β*_.

Markov chain Monte Carlo (MCMC) methods were used to sample the posterior distributions of the parameters of interest. For each of the competing models, we ran three chains in parallel using package dclone (Solymos, 2010) with different sets of initial values. The first 10,000 MCMC samples were discarded, known as the burn‐in period, after having checked that convergence was satisfactory. Convergence was visually assessed using sample path plots in conjunction with the Brooks–Gelman–Rubin diagnostic $\hat{r}$ (Brooks & Gelman, 1998), with values close to 1.00 indicating adequate convergence. Chains then ran for 100,000 iterations after burn‐in, and a total of 3000 MCMC samples (every 100th sample of each chain) were used for inference. We determined that a covariate had an effect if a 95% credible interval (CRI) of the posterior distribution of that parameter did not include 0. We assessed support for inclusion of weaning length using a measure of out‐of‐sample predictive ability of each model, the Widely Applicable Information Criterion (WAIC, Watanabe, 2010), where a model with a smaller WAIC is judged a better fit.

## 3 RESULTS

**FIGURE 1:**
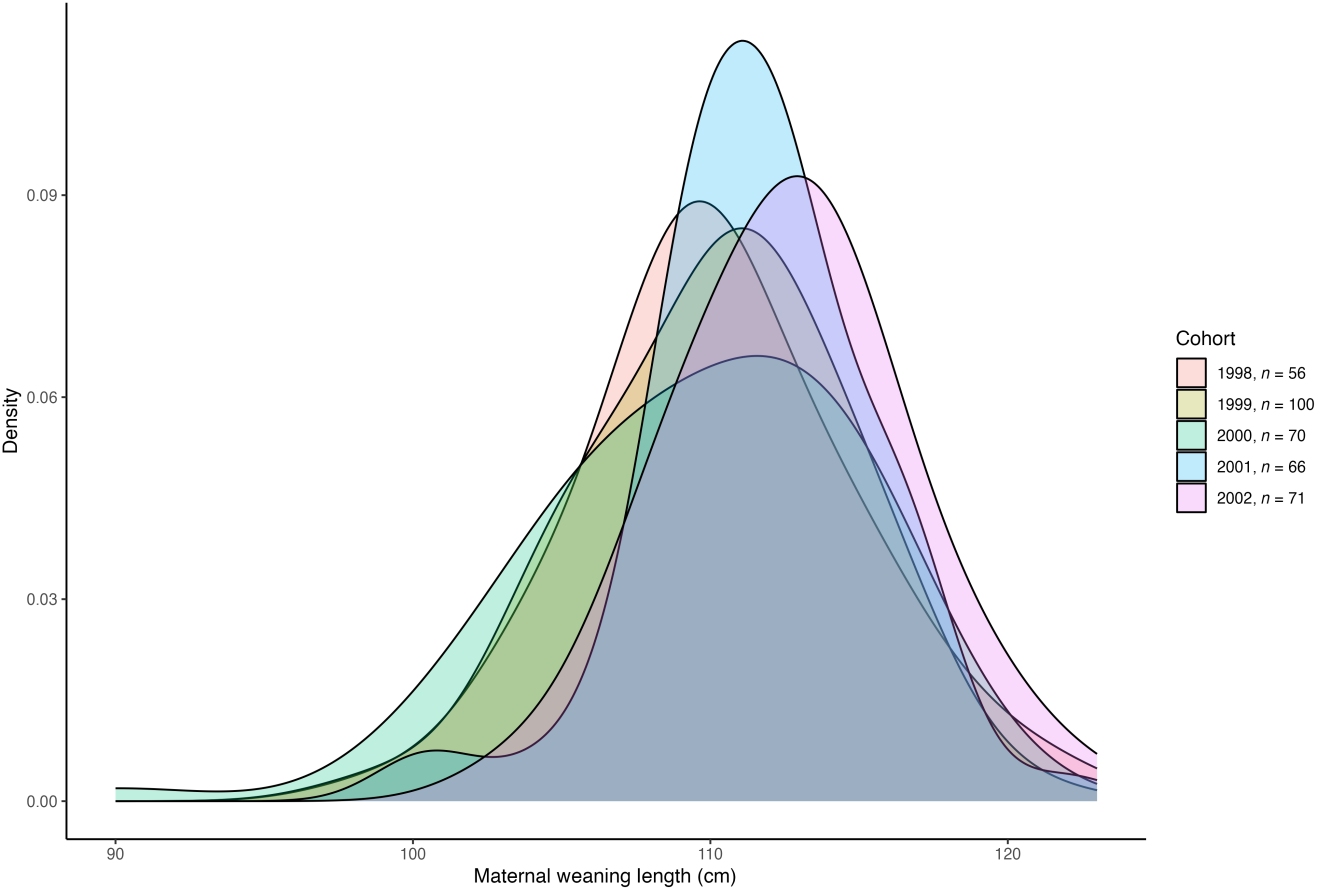
Density plots of the distribution of weaning lengths of our sample of females by cohort, 1998–2002.

We analyzed the reproductive histories of 363 females born from 1998 to 2002 that gave birth to a total of 3457 pups. 2.5% (9/363) of those females recruited to the breeding population at age 4, 31.4% (114/363) had their first birth at the age of 5, 24.5% (89/363) at the age of 6, and 30.5% (111/363) recruited after age 6. From primiparity to the most recent year of the study, 2020, females had an average of 10 pups (SE = 4.48, ranging 1–17). These females' weaning lengths (*L*_*w*_), ranged from 90 to 132 cm, with an average of 112.7 cm (SE = 4.28). We did find a cohort effect on *L*_*w*_ (ANOVA, *p* = .003), where females born in 2002 that recruited to the breeding population had significantly longer *L*_*w*_ than other cohorts analyzed (Tukey HSD, Figure 1).

### 3.1 Effect of weaning length on future reproductive performance

**FIGURE 2:**
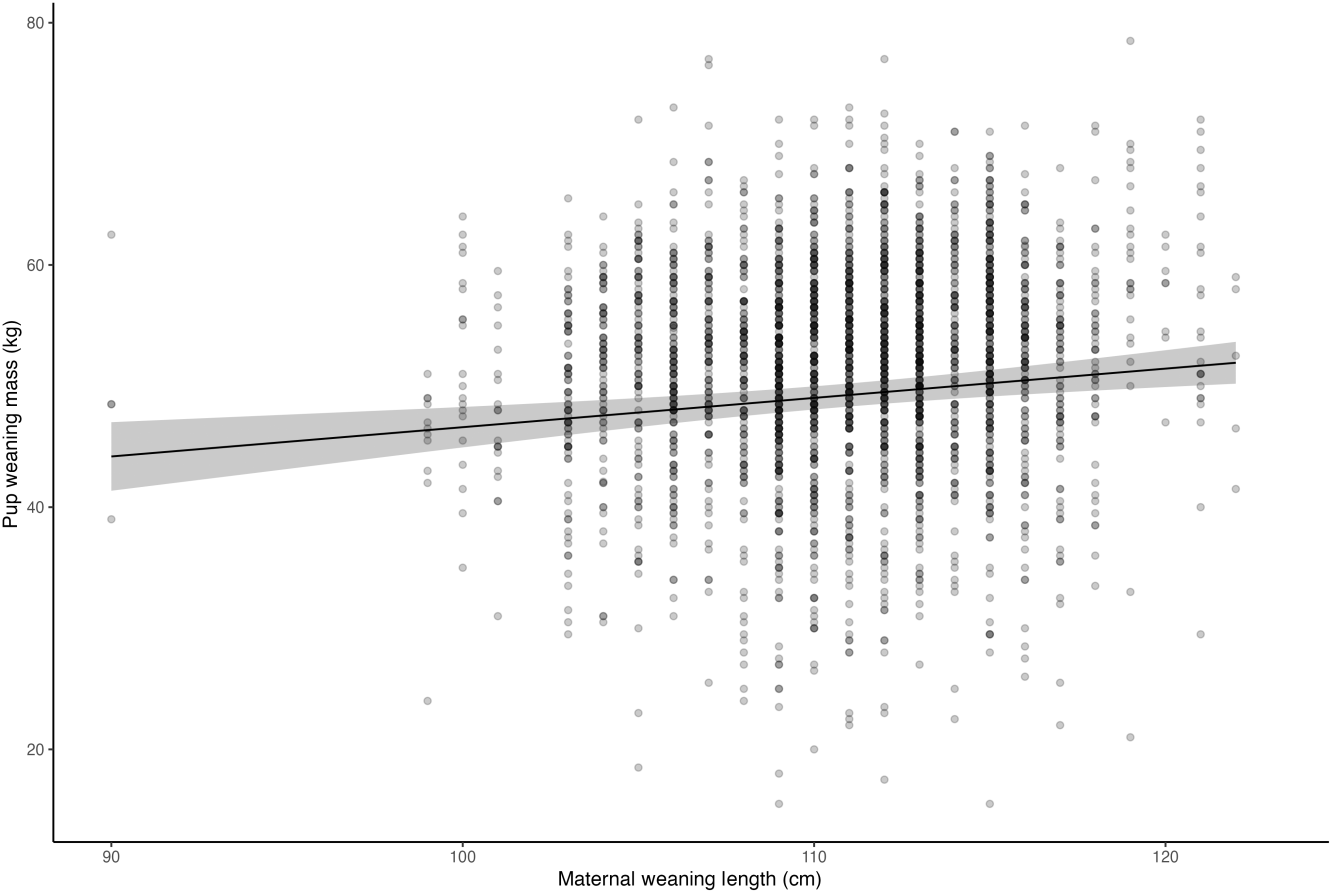
Estimated effect of an individual's weaning length on their future provisioning performance, controlling for the effects of age, sex, parity, year, and individual effects not accounted for by weaning length.

**FIGURE 3:**
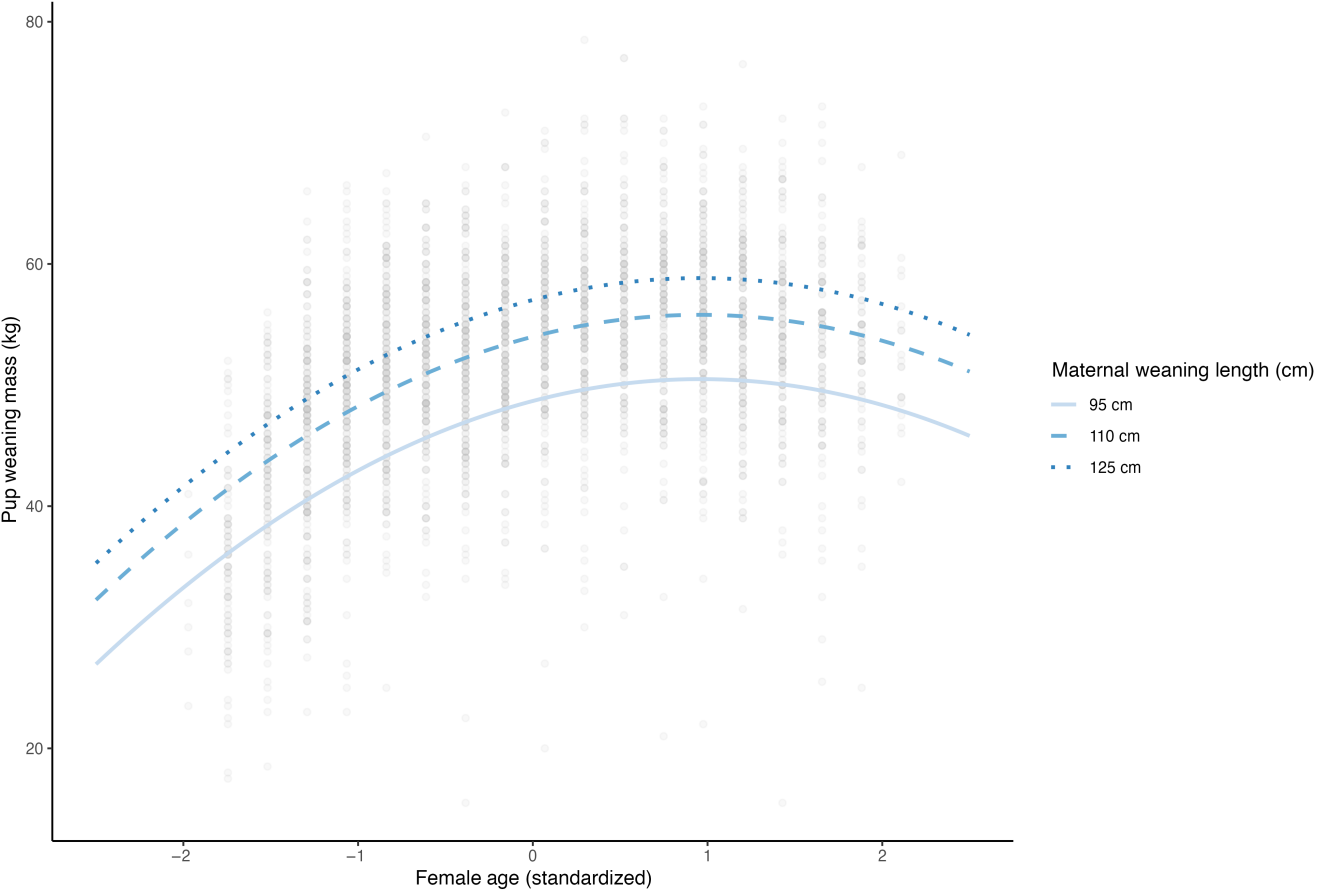
Estimated effect of weaning length on provisioning performance as a female ages. Lines are 0.025%, 50%, and 97.5% quantiles of weaning lengths corresponding to 95, 110, and 125 cm.

**FIGURE 4:**
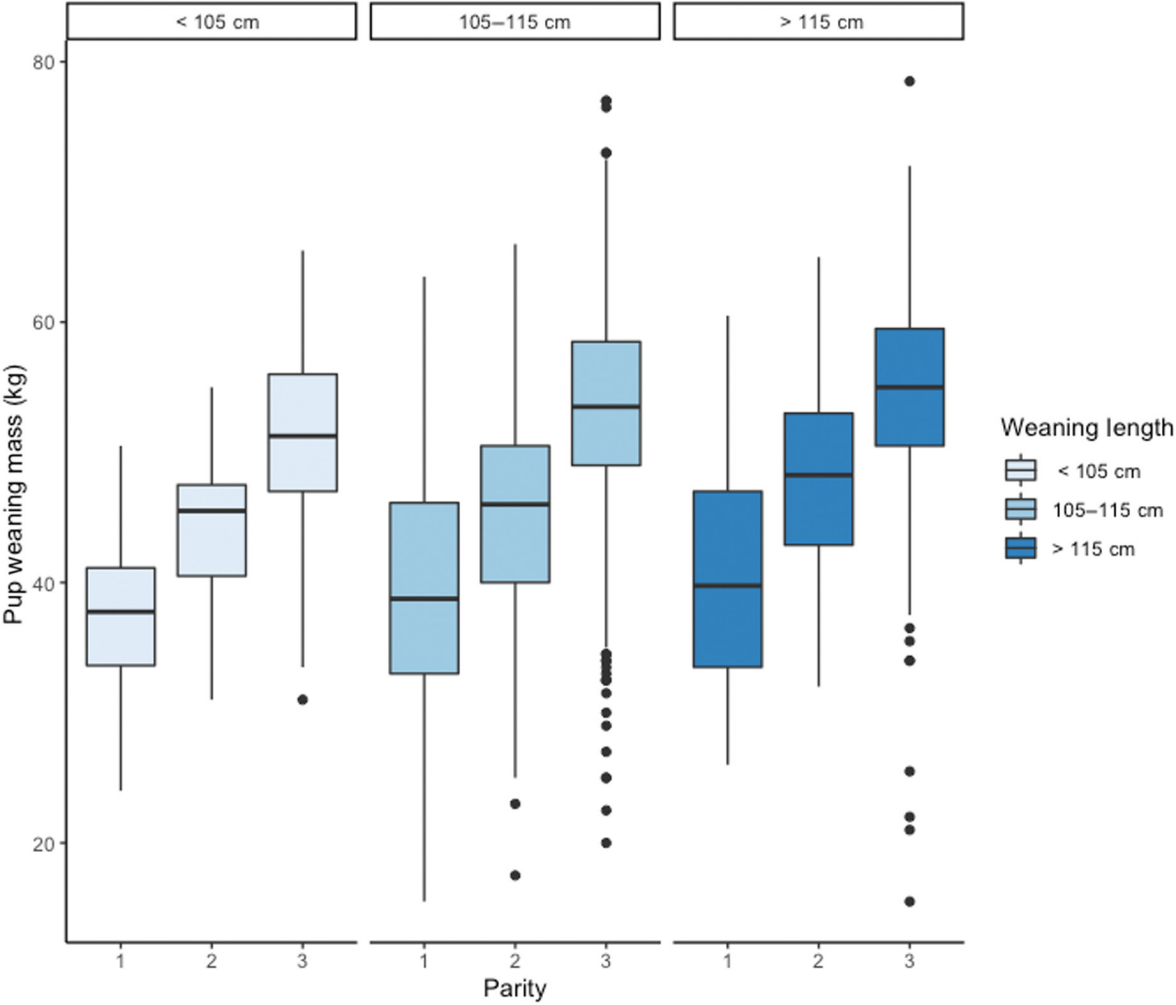
There is no evidence for an interactive effect of weaning length and parity–effect of weaning length on pup weaning mass does not taper off (*p* > .05, Table 1). Boxplots of pup weaning masses for individuals with short (90–105 cm), average (105–115 cm), and tall (115–132 cm) weaning lengths (panels) over the 1st, 2nd, and 3+ parities.

**FIGURE 5:**
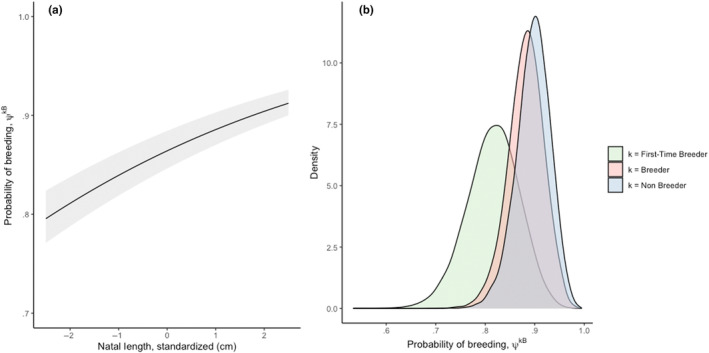
Results from the Markov chain multistate model describing probability of breeding, *ψ* ^*kB*^, as a function of (a) weaning length, and (b) the female's previous state in year *t* − 1.

*L*_*w*_ was positively associated with a female's future provisioning performance (*p* < .001, Table 1, Figures 2 and 3). The best supported model describing pup weaning masses included an additive, linear effect of weaning length as a covariate, though there was also modest support for a quadratic effect (Table 1, Appendix A: Table A2). Females who had the longest weaning lengths (132 cm) proceeded to give birth to offspring that weaned 8 kg heavier, on average, than conspecifics who had the shortest weaning lengths (90 cm, Table 3, Figures 2 and 3). Although we expected weaning body length to have a greater effect on early parities (such that the effect weakened over time), we found no support for an interactive model between *L*_*w*_ and parity (Table 1, Figure 4, Appendix A: Table A3). Repeatable differences among individuals accounted for 41% of the variance in pup weaning mass. Year accounted for only 10.8% of the variance in weaning mass, suggesting that among‐year environmental effects were small. Weaning length was also positively associated with a female's future reproductive rate. Model output from fitted multistate Markov models estimated that weaning length accounts for the spread in annual reproductive probability to range from 0.715 for females who had the shortest *L*_*w*_ to 0.916 for females who had the longest *L*_*w*_ (*L*_*w*_ range = 90–132 cm, Table 4, Figure 5). Model fits displayed no evidence of inadequate convergence to stationary distributions.

**TABLE 3:** Parameter estimates for favored linear mixed effects model describing variation in pup weaning mass as a function of maternal age, experience (parity), pup sex, weaning length *L*_*w*_, and random effects of year and individual.

Parameter	Mean	SE
Intercept	49.22	0.791
*π*_1_	14.46	1.26
*π*_2_	−11.88	1.19
*π*_3_|*par*_*i*,*t*_ = 2	4.01	0.55
*π*_3_|*par*_*i*,*t*_ = 3	6.23	0.63
*π*_4_	−2.26	0.22
*π*_5_	1.07	0.29
$\sigma_{\mathrm{ID}}^{2}$	4.67	
$\sigma_{\text{year}}^{2}$	1.29	
$\sigma_{\text{residual}}^{2}$	5.45	

**TABLE 4:** Posterior mean, SD, 2.5%, 50%, and 97.5% quantiles, and convergence diagnostic $\hat{r}$ of parameters for preferred multistate model, describing variation in reproductive rate ($\psi_{i,t}^{\mathrm{kB}}$) as a function of previous reproductive state (B = breeder, F = first‐time breeder, and N = nonbreeder), quadratic effect of maternal age (*λ*_1_, *λ*_2_), linear maternal length as young *L*_*w*_ (*λ*_5_), and random effects of individual and year. The effect of previous state is reported here as transition rates among F, B, and N for ease of interpretation.

Parameter	$\hat{r}$	Mean	SD	2.5%	50%	97.5%
$\psi_{i,t}^{\mathrm{BB}}$	1.003	0.861	0.038	0.784	0.861	0.935
$\psi_{i,t}^{\mathrm{FB}}$	1.003	0.779	0.056	0.665	0.780	0.888
$\psi_{i,t}^{\mathrm{NB}}$	1.003	0.878	0.037	0.803	0.879	0.950
*λ*_1_	1.016	0.385	0.083	0.125	0.412	0.463
*λ*_2_	1.016	−0.355	0.083	−0.440	−0.380	−0.096
*λ*_5_	1.001	0.549	0.020	0.513	0.549	0.594
*p*	1.007	0.975	0.021	0.924	0.980	0.999
$\sigma_{\beta }^{2}$	1.006	0.895	0.151	0.673	0.869	1.269
$\sigma_{\theta }^{2}$	1.001	1.310	0.695	0.371	1.169	3.036

### 3.2 Cost of reproduction in breeding rate

In this analysis fit to the reproductive data of individuals from the 1998 to 2002 cohorts, the fitted multistate model estimated somewhat (~2%) higher reproductive probabilities for individuals that did not breed in the previous year (Table 4). However, previous analyses on a larger subset of this population including individuals born in the 1960s–1980s, did not find evidence for a cost of reproduction expressed in reproductive rate. In one of these previous analyses, individuals that reproduced in the current year were on average 11% more likely to breed the next year than those that skipped reproduction (Badger et al., 2020; den Heyer & Bowen, 2017, Figure 6). Importantly, females born in the 1960s–1980s recruited during a period of exponential growth with population densities much lower than the females recruiting in the present study (den Heyer & Bowen, 2017). The result of this current analysis, indicating a slight cost under higher population densities, contrasting with the previous studies indicating no cost when population densities were lower suggest that the cost of reproduction may only be expressed at higher population densities.

**FIGURE 6:**
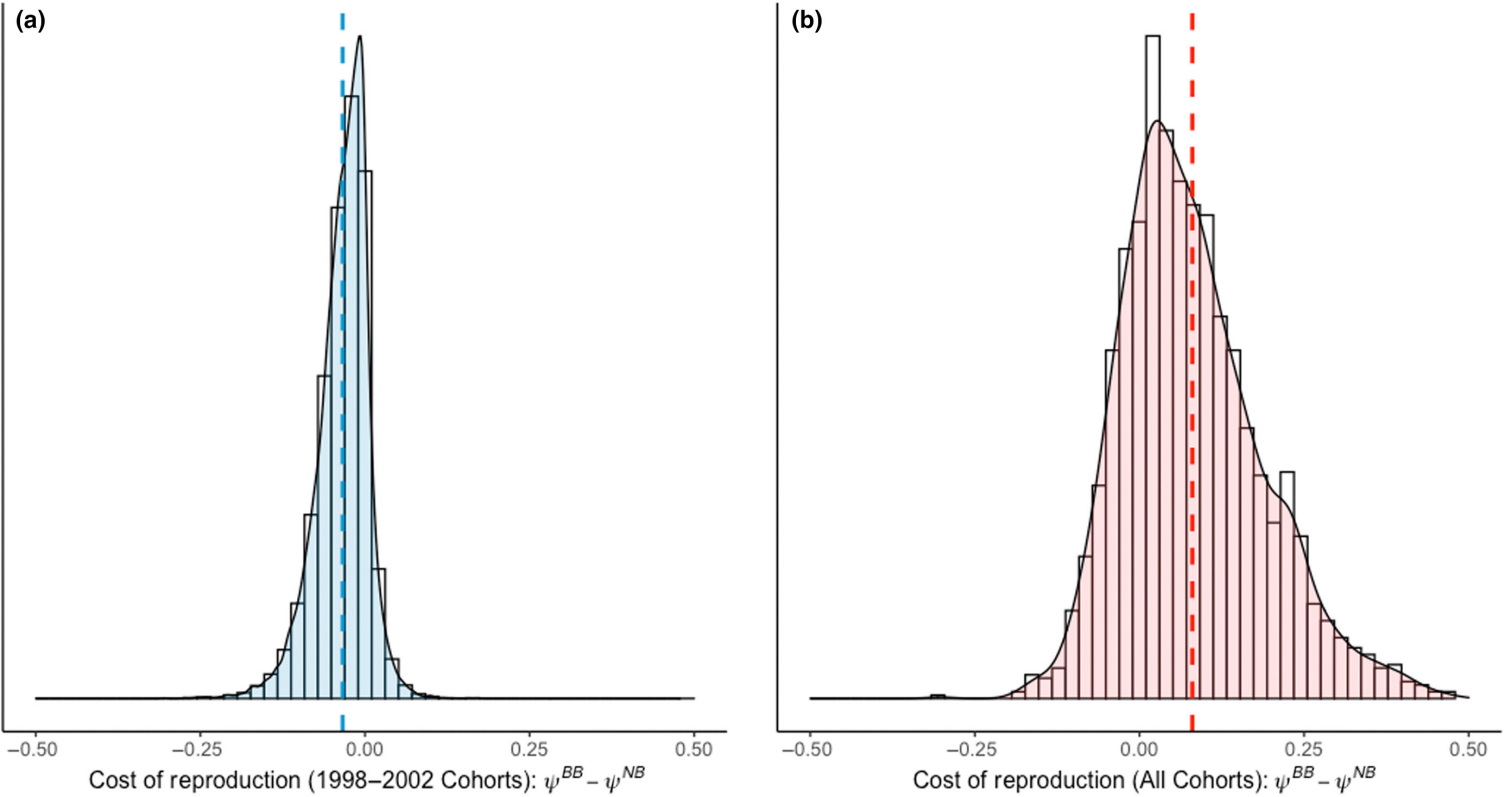
Cost of reproduction is estimated by finding the difference between reproductive probabilities of nonbreeders and breeders: panels depict posterior distribution of *ψ* ^*BB*^ (breeder to breeder) minus posterior distribution of *ψ* ^*NB*^ (nonbreeder to breeder) for (a) output of the preferred model reported here, estimating reproductive probabilities for females born from 1998 to 2002, and (b) the output from (Badger et al., 2020), a similar model estimating reproductive probabilities for females born 1962, 1969, 1970, 1973, 1974, 1985–87, 1989, and 1998–2002. Note that for (b), the models did not estimate a cost of reproduction in terms of reproductive rate, where *ψ* ^*BB*^ > *ψ* ^*NB*^, i.e. current reproduction does not incur a “penalty” to future reproduction. By contrast, our sample of females (a) show a slight cost of reproduction *ψ* ^*BB*^ < *ψ* ^*NB*^, where individuals are slightly more likely to breed in a given year if they had skipped reproduction previously.

### 3.3 Cohort effects in reproductive performance

After detecting a cohort effect in (*L*_*w*_), we incorporated cohort effects into reproductive performance models of breeding rate and offspring mass. Individuals from the 2002 cohorts had lighter pups on average than individuals from other cohorts (Appendix A: Table A4), though this model performed worse in out‐of‐sample predictive accuracy than models without cohort effects (Table 1). In reproductive rate, our multistate models also estimated a lower breeding rate of individuals born in the 2002 cohort (Appendix A: Table A4) relative to other cohorts analyzed. However, this model also performed poorly relative to models not including cohort as a covariate (Table 2). We further caution the interpretation of the result of this multistate model including cohort effects as we were not able to control for the effects of maternal age due to issues with convergence likely stemming from multicollinearity of the age, cohort, and parity variables.

## 4 DISCUSSION

We found positive covariation between an individual's weaning length and subsequent adult reproductive performance measured by two traits from a large sample of gray seals observed for over 20 years. Mothers with the longest weaning lengths produced pups nearly 8 kg heavier and were 20% more likely to breed in a given year than mothers with the shortest weaning lengths. This result is consistent with a “bigger is better” hypothesis (Bowen et al., 2006), in which longer offspring mature to have higher reproductive success. However, as weaning length is only weakly correlated with adult length, the pattern is not simply the result of larger adults being more fit. Instead, weaning length appears to act as a carry‐over effect of juvenile morphology on lifetime reproductive success.

The observed spread in offspring size and reproductive frequency should drive substantial variation in lifetime reproductive output. Badger et al. (2020) found reproductive frequency and the probability of weaning a viable pup were highly correlated within individual gray seal females, and over their lifetimes higher performing females will average 1.83 times more successful pups than poorer performers. Furthermore, for weaning masses below the population mean (51.5 kg), pup survival is dependent on mass (Bowen et al., 2015; Hall et al., 2001), with each 1 kg decrease below average corresponding to a 0.12 decrease in survival to reproductive recruitment (on the logit scale). Consequently, an 8 kg spread would have a large impact on the probability a female's offspring will reach sexual maturity, affecting both maternal and offspring fitness.

### 4.1 Implications for maternal fitness

Our findings show that gray seal mothers increase maternal fitness by producing longer pups, as longer pups mature to be more productive mothers. While the effect of offspring body size on maternal fitness has been extensively studied (Cody, 1966; Krist, 2011; Lack, 1947; Pettersen et al., 2015; Rollinson & Hutchings, 2013; Smith & Fretwell, 1974; Stearns, 2000), mass or fat reserves, and not length, are the typical measure of size. This methodological bias is likely due to the difficulty of accurately measuring length; body posture can greatly impact length measurements, while mass is accurately and precisely measured with a calibrated scale. Body length and fat reserves of offspring, however, reflect different aspects of maternal quality; larger skeletal size is more likely to have a significant heritable component while stored energy is a measure of maternal effort and investment. Provisioning offspring with large energy reserves requires considerable reproductive energy expenditure in both acquisition of resources (e.g., foraging efficiency, prey choice, and instraspecific competition) and effectively transferring resources to offspring (e.g., lactation efficiency, nursing behavior). Some maternal behaviors are likely to have a genetic basis (Bubac et al., 2021), but increasing an offspring's skeletal size likely has a relatively larger heritable genetic component. The genetic basis of skeletal architecture is unknown for pinnipeds, but divergent selection experiments in domestic mammals suggest that dozens to thousands of loci underlie variation in structural body size (Kemper et al., 2012).

Allocation theory predicts an asymptotic relationship on an offspring's size and its survival because parents receive decreasing returns on investment in offspring fitness after a certain point (Smith & Fretwell, 1974). Previous analyses of this population suggest stabilizing selection on weaning mass, where offspring survival to recruitment levels out near the average weaning mass and slowly decreases at increasingly higher weaning masses (Bowen et al., 2015). In contrast, body length appears to be subject to directional selection (at least in the current ecological environment), evidenced by a monotonically increasing relationship between body length and offspring survival to reproductive recruitment (Bowen et al., 2015). Early growth rate will vary among individuals as a function of their genetic makeup, environmental conditions, and an individual's foraging success in those conditions (Harrison et al., 2011; Madsen & Shine, 2000). While fat reserves provide crucial resources during the transition to independent foraging, fatter pups are likely more buoyant, which in diving animals would result in less efficient foraging and greater vulnerability to predation (Hindell et al., 1999; Sogard, 1997). Longer individuals, however, may gain a tangible benefit throughout early stages due to greater swimming speed, diving ability, and less vulnerability to predators, which may be accentuated in the current highly competitive foraging environment (Breed et al., 2013). The possible mechanisms driving relationships between early traits and survival remain to be tested, but results from this analysis indicate benefits of length have a persistent effect on fitness and potential for strong transgenerational effects on reproductive output.

### 4.2 Weaning length as a source of individual variation in quality

Recent analyses of this population indicate substantial differences in quality (i.e., lifetime reproductive success) among individuals (Badger et al., 2020). Although it is expected that maternal effects on offspring size are most significant in early life (Dias & Marshall, 2010), with compensatory growth or other factors reducing impact later in life (e.g., domestic sheep, Wilson & Réale, 2006, red squirrels, Wauters et al., 1993), our results suggest variation in early body length may explain some of the observed variation in individual quality across an individual's lifetime. Individuals that were longer as young juveniles consistently outperform those of shorter lengths in both survival to sexual maturity (Bowen et al., 2015) and reproductive success once recruited (this study).

Effective acquisition and conservation of food energy is impacted by morphological traits such as body length, and potentially drive substantial variation in reproductive success. Although larger animals have higher absolute metabolic requirements, larger individuals exhibit lower mass‐specific rates of metabolism which confers a suite of physiological and ecological benefits at greater body sizes (Gearty et al., 2018; Glazier, 2005; Kleiber, 1947). These advantages include a low cost of transport, enhanced fasting ability, and, for animals such as seals, the ability to make longer and deeper foraging dives (Costa, 1993; Peters, 1983).

The extent to which body length, independent of mass, may offset the energetic cost of foraging is, however, unknown in many systems, including gray seals. In Weddell seals, Wheatley et al. (2006) found that postpartum mass of shorter females was significantly lower in years of poor environmental conditions whereas the mass of longer females did not differ between years. This suggested shorter females were less successful foragers than their larger conspecifics and may generally be more susceptible to environmental variation (Wheatley et al., 2006). If longer females are more successful foragers, or more robust to environmental variation, they would have a distinct advantage in accumulating and storing energy needed for reproduction.

Alternatively, length may be advantageous in growing juvenile stages for gray seals, but attenuate over time as they grow. Large skeletal size as an adult could also be subject to stabilizing selection, where longer individuals experience different physical constraints and energetic costs that cause impairment relative to shorter animals (Williams et al., 2000). Increasing body size will increase costs to sustaining body condition and maintaining buoyancy in the water column. Although smaller animals have a higher mass‐specific metabolism, their absolute energy requirements are lower (Costa, 1993, Peters, 1983) and so could be less vulnerable to food scarcities. Smaller prey items are relatively unprofitable to larger individuals than smaller individuals, requiring additional costly prey captures to reach energy requirements, decreasing the efficiency of a foraging bout (Costa, 1993) and competitive ability under resource limitation (Clutton‐Brock, 1988). The size spectra of prey of some ecological environments may be distinctly unfavorable to the largest individuals; profitably sized prey may not be available to larger individuals, where smaller individuals can forage efficiently on smaller prey that are more abundant.

Our finding that longer pups do not necessarily mature into longer adults suggests that selection against very large size in adult females may be present. Bowen et al. (2015) found a positive, but weak correlation between body length of these female pups and their length at primiparity (age at first reproduction), and length data collected sporadically since suggests the relationship between weaning and adult length is weak through adulthood. Weaning body length accounted for 6% of the variation in primiparous length (*n* = 325, Bowen et al., 2015), 4.6% of variation in body length of adult females during early adulthood (5–10 years, *n* = 268, W. Don Bowen, Cornelia E. den Heyer, unpublished data) and 4.3% of the variation in body length of older females (10+ years, *n* = 29, W. Don Bowen, Cornelia E. den Heyer, unpublished data). Consequently, it is unlikely our results are due simply to longer juveniles remaining long throughout life. Growth and reproduction are involved in a classic physiological trade‐off, and further somatic investment during reproductive years may not maximize fitness (Clutton‐Brock, 1984; Green & Rothstein, 1991; Partridge & Harvey, 1988; Stearns, 1992; van Noordwijk & de Jong, 1986).

### 4.3 Carry‐over effects of early life morphology

The covariation between weaning length and future reproductive performance likely acts as a carry‐over effect, with larger size at weaning permitting better growth and self‐maintenance as a juvenile. This better performance as a juvenile translates into greater adult performance, rather than larger size granting the same relative advantage throughout life. Carry‐over effects describe how the environment experienced early in life affect the expression of traits in subsequent life stages or in habitats (Moore & Martin, 2019; O'Connor et al., 2014). Carry‐over effects that occur at the individual level can affect a wide range of fitness parameters. They result in long‐term, large‐scale consequences on a population's dynamics and composition and so influence multiple levels of biological organization from individuals, populations, and even community structures (Betini et al., 2013; Moore & Martin, 2019; Norris, 2005; O'Connor et al., 2014).

Carry‐over effects linking ecological conditions experienced early in life to later performance are well‐documented (Descamps et al., 2008; Garcia et al., 2019; Gratton & Denno, 2003; Harrison et al., 2011; Madsen & Shine, 2000; Marshall et al., 2006; Moore & Martin, 2019; Nussey et al., 2007; O'Connor et al., 2014), though such demonstrations are relatively rare for long‐lived mammals (Coltman et al., 1999; Festa‐Bianchet et al., 2000; Nussey et al., 2007). Food availability during early development is understood to be a key environmental factor driving carry‐over effects (Descamps et al., 2008; Harrison et al., 2011), with the ultimate driver being habitat quality, or less commonly reported, intraspecific density.

Our results suggest female gray seals experience a carry‐over effect of their early life morphology on future reproductive performance, that may ultimately be driven by negative density dependence. In a competitive environment, longer individuals outperform shorter conspecifics early in life, and the advantages of this early life performance persist through life, even where the actual size differences do not. Breed et al. (2013) documented that juvenile gray seals may be competitively excluded from key foraging grounds by adult females in the current highly competitive environment, potentially contributing to the stark decline in juvenile apparent survival in the 1998–2002 cohorts (den Heyer et al., 2013) This exclusion may continue into adulthood, such that there is further intense competition to secure ideal foraging grounds. Longer juveniles may be more able to compete with adults and secure better foraging habitat, which carry over into reproductive years affecting their reproductive fitness traits (Lloyd et al., 2019).

### 4.4 Implications for population dynamics

The Sable Island gray seal colony has increased dramatically over the past 60 years with near maximum population growth of 13% per year between the 1960s and late 1990s (Bowen, 2011) and a reduced rate of increase of 4% from 1997 to 2016 (den Heyer et al., 2017, 2021). Female gray seals born during the exponential growth of the 1980s and 1990s had apparent survival probabilities of 0.7–0.8 (den Heyer et al., 2013). By contrast, in the late 1990s to early 2000s, when our study animals were born, the population had entered a period of reduced population growth as it seemingly approached carrying capacity (Bowen, 2011; Bowen et al., 2007; den Heyer et al., 2017, 2021), with drastically reduced apparent juvenile survival probabilities ranging from 0.26 to 0.39. Previous analyses suggest a size‐selective mortality, where individuals with longer weaning lengths were more likely to reach sexual maturity (Bowen et al., 2015).

In the analysis presented here, our sampling scheme and modeling framework likely yield a conservative estimate of the relationship between weaning length and reproductive performance, as we only included individuals that survived to breeding age and (1) were observed in at least two breeding seasons and (2) nursed their pup long enough to be recorded by our research teams. These constraints result in a sample that explores the relatively better performing regions of the spectrum of reproductive investment. Inexperienced or low‐quality mothers may frequently flee or abandon pups, and these reproductive attempts would not be recorded in our observations (though this is not a major source of bias, see Hammill et al., 2017). For these reasons, the poorest performers are less likely to be observed, resulting in a slightly larger proportion of high‐quality females in our sample than present in the Sable Island breeding population.

Our sample of females also make up the postselection distribution of body size, and this study can perhaps be viewed as a lens into the reproductive performance of individuals growing under intense selection pressure and slowing population growth (Allen et al., 2008; Coltman et al., 1999). In addition to our results linking early size with reproductive success, we found that this sample of females exhibited a slight cost of reproduction not detected when a larger subset of the population was analyzed by Badger et al. (2020). That sample included females born in the 1960s, 1970s, and 1980s that were juveniles when population densities were much lower. From this, we infer that ecological conditions during early stages can mediate future trade‐offs and shape the natural selection on life history and pace‐of‐life (Clutton‐Brock et al., 1987; Coltman et al., 1999). Intensified competition among these age groups may drive a less favorable energetic trade‐off between survival and supporting reproduction for individuals recruiting into an intensely competitive environment.

### 4.5 Implications and conclusions

Here, we found that body size when young was positively associated with two measures of reproductive performance later in life, and because early size is only weakly related to adult size, this relationship appears to be acting as a carry‐over effect. Our findings underscore the multiple lines of evidence before us that have demonstrated that maternal fitness depends on attributes of offspring size and their cascading effects on offspring fitness, and constitute the first documentation of size carry‐over effects of early ontogeny on adult performance in marine mammals. In this case, early body size appears to be acting as a carry‐over effect coinciding with shifting population dynamics and increasing negative density dependence.

Our findings prompt further investigation into how negative density dependence shapes the evolution of life histories and morphology in a long‐lived, iteroparous animal. Phenotypic selection across life stages will vary according to how fitness is maximized in a given environment, and will have large‐scale consequences in ecological and evolutionary time scales. As long‐lived iteroparous mammals must allocate their reproductive effort over many years to maximize fitness, parental genotypes that produce longer offspring lengths may provide a fitness advantage. Significant heritability of length traits have been estimated in many systems (e.g., hindleg length Soay sheep, Wilson et al., 2007) though the extent of heritability in body length in gray seals (and indeed seals and marine mammals generally) has not been tested. In the gray seal cohorts we studied, there is evidence for positive selection for weaning length in recruitment (Bowen et al., 2015), and the results reported here indicate that weaning length continues to correlate with markers of fitness after recruitment in more frequent breeding and higher investment in pups. Although this directional selection is predicted to, if heritable, cause longer weaning body lengths to evolve over time, it remains untested whether the population overall is getting longer as selection pressures from increasing seal density intensify, or if there is counteracting selection against extremely long adults even when longer weaning lengths are favored. Gray seals have particularly high and consistent survival as adults (0.989 ± 0.001 for females aged 4–24, 0.901 ± 0.004 for females aged 25+, den Heyer & Bowen, 2017), so directional selection on body length as adults is more likely to act through variation in reproductive performance. Further investigation into changes in size‐selective vital rates as the population continues to increase would likely yield important insights into density‐related evolutionary changes in long‐lived animals.

## AUTHOR CONTRIBUTIONS

**Janelle J. Badger:** Conceptualization (equal); formal analysis (lead); funding acquisition (supporting); project administration (equal); writing – original draft (lead). **W. Don Bowen:** Conceptualization (equal); data curation (equal); funding acquisition (equal); methodology (lead); writing – review and editing (equal). **Cornelia E. den Heyer:** Data curation (equal); funding acquisition (equal); methodology (equal); writing – original draft (equal). **Greg A. Breed:** Conceptualization (equal); funding acquisition (equal); project administration (equal); supervision (lead); writing – review and editing (equal).

## FUNDING INFORMATION

This work is supported by the National Science Foundation Graduate Research Fellowship Program under Grant No. 1839290 awarded to JJB. Any opinion, findings, and conclusions or recommendations expressed in this material are those of the authors and do not necessarily reflect the views of the National Science Foundation. Data collection was supported by the Department of Fisheries and Oceans Canada, and Natural Sciences and Engineering Research Council grants to S. J. Iverson and W. D. Bowen and the Department of Fisheries and Oceans Centre of Expertise for Marine Mammalogy.

## CONFLICT OF INTEREST STATEMENT

We declare we have no competing interests.

## Data Availability

The Department of Fisheries and Oceans Canada scientific data are a public resource and subject to full and open access within 2 years of being acquired or generated. Please refer all data enquiries directly to the DFO.

## APPENDIX A

### A.1

Model results for different formulations of the linear mixed effects model describing variation in pup weaning mass.

**TABLE A1:** Parameter estimates for Mod 0, a null linear mixed effects model describing variation in pup weaning mass as a function of maternal age (*π*_1_, *π*_2_), female experience (parity, *π*_3_), pup sex (*π*_4_), and random effects of year and individual.

Parameter	Mean	SE
Intercept	49.15	0.75
*π*_1_	14.49	0.22
*π*_2_	−11.97	1.19
*π*_3_|*par*_*i,t*_ = 2	4.05	0.55
*π*_3_|*par*_*i,t*_ = 3	6.26	0.62
*π*_4_	−2.26	0.22
$\sigma_{\mathrm{ID}}^{2}$	4.77	
$\sigma_{\text{year}}^{2}$	1.28	
$\sigma_{\text{residual}}^{2}$	5.45	

*Note*: Refer to Table 1 for model structure.

**TABLE A2:** Parameter estimates for Mod 2, a linear mixed effects model describing variation in pup weaning mass as a function of maternal age (*π*_1_, *π*_2_), female experience (parity, *π*_3_), pup sex (*π*_4_), a quadratic effect of natal length (*π*_5_, *π*_6_), and random effects of year and individual.

Parameter	Mean	SE
Intercept	48.79	0.75
*π*_1_	14.45	1.26
*π*_2_	−11.87	1.20
*π*_3_|*par*_*i,t*_ = 2	4.01	0.55
*π*_3_|*par*_*i,t*_ = 3	6.23	0.62
*π*_4_	−2.26	0.22
*π*_5_	−10.82	8.83
*π*_6_	11.49	8.52
$\sigma_{\mathrm{ID}}^{2}$	4.66	
$\sigma_{\text{year}}^{2}$	1.29	
$\sigma_{\text{residual}}^{2}$	5.50	

*Note*: Refer to Table 1 for model structure.

**TABLE A3:** Parameter estimates for Mod 3, a linear mixed effects model describing variation in pup weaning mass as a function of maternal age (*π*_1_, *π*_2_), maternal experience (parity, *π*_3_), pup sex (*π*_4_), natal length (*π*_5_) an interactive effect of natal length and parity (*π*_6_), and random effects of year and individual.

Parameter	Mean	SE
Intercept	49.22	0.75
*π*_1_	14.51	1.26
*π*_2_	−11.92	1.20
*π*_3_|*par*_*i,t*_ = 2	4.01	0.55
*π*_3_|*par*_*i,t*_ = 3	6.24	0.62
*π*_4_	−2.26	0.22
*π*_5_	0.93	0.45
*π*_6_|*par*_*i,t*_ = 2	0.47	0.52
*π*_6_|*par*_*i,t*_ = 3	0.12	0.40
$\sigma_{\mathrm{ID}}^{2}$	4.67	
$\sigma_{\text{year}}^{2}$	1.29	
$\sigma_{\text{residual}}^{2}$	5.46	

*Note*: Refer to Table 1 for model structure.

**TABLE A4:** Parameter estimates for Mod 4, a linear mixed effects model describing variation in pup weaning mass as a function of maternal age (*π*_1_, *π*_2_), female experience (parity, *π*_3_), pup sex (*π*_4_), natal length (*π*_5_), female cohort (*π*_6_), and random effects of year and individual.

Parameter	Mean	SE
Intercept	48.78	1.00
*π*_1_	14.49	1.26
*π*_2_	−11.95	1.20
*π*_3_|*par*_*i,t*_ = 2	3.98	0.55
*π*_3_|*par*_*i,t*_ = 3	6.18	0.62
*π*_4_	−2.26	0.22
*π*_5_	1.15	0.29
*π*_6_|*cohort*_*i*_ = 1999	0.80	0.89
*π*_6_|*cohort*_*i*_ = 2000	1.11	0.97
*π*_6_|*cohort*_*i*_ = 2001	0.27	1.00
*π*_6_|*cohort*_*i*_ = 2002	−0.09	1.02
$\sigma_{\mathrm{ID}}^{2}$	4.67	
$\sigma_{\text{year}}^{2}$	1.29	
$\sigma_{\text{residual}}^{2}$	5.45	

*Note*: Refer to Table 1 for model structure.
